# Strong immune responses and protection of PcrV and OprF-I mRNA vaccine candidates against *Pseudomonas aeruginosa*

**DOI:** 10.1038/s41541-023-00672-4

**Published:** 2023-05-25

**Authors:** Xingyun Wang, Cong Liu, Nino Rcheulishvili, Dimitri Papukashvili, Fengfei Xie, Jiao Zhao, Xing Hu, Kaiwei Yu, Nuo Yang, Xuehua Pan, Xueyan Liu, Peng George Wang, Yunjiao He

**Affiliations:** 1 Department of Pharmacology, School of Medicine, Southern University of Science and Technology, Shenzhen, China; 2 Department of Critical Medicine, Shenzhen People’s Hospital, First Affiliated Hospital of Southern University of Science and Technology, Second Clinical Medicine College of Jinan University, Shenzhen, China

**Keywords:** RNA vaccines, Vaccines

## Abstract

*Pseudomonas aeruginosa* (PA) is a leading cause of hospital-acquired and ventilator-associated pneumonia. The multidrug-resistance (MDR) rate of PA is increasing making the management of PA a global challenge. Messenger RNA (mRNA) vaccines represent the most promising alternative to conventional vaccines and are widely studied for viral infection and cancer immunotherapy while rarely studied for bacterial infections. In this study, two mRNA vaccines encoding PcrV– the key component of the type III secretion system in *Pseudomonas* and the fusion protein OprF-I comprising outer membrane proteins OprF and OprI were constructed. The mice were immunized with either one of these mRNA vaccines or with the combination of both. Additionally, mice were vaccinated with PcrV, OprF, or the combination of these two proteins. Immunization with either mRNA-PcrV or mRNA-OprF-I elicited a Th1/Th2 mixed or slighted Th1-biased immune response, conferred broad protection, and reduced bacterial burden and inflammation in burn and systemic infection models. mRNA-PcrV induced significantly stronger antigen-specific humoral and cellular immune responses and higher survival rate compared with the OprF-I after challenging with all the PA strains tested. The combined mRNA vaccine demonstrated the best survival rate. Moreover, the mRNA vaccines showed the superiority over protein vaccines. These results suggest that mRNA-PcrV as well as the mixture of mRNA-PcrV and mRNA-OprF-I are promising vaccine candidates for the prevention of PA infection.

## Introduction

*Pseudomonas aeruginosa* (PA) is an aerobic/facultative anaerobic, gram-negative, non-spore-forming rod bacterium, which poses a serious threat to global public health^1,2^. As a prevalent opportunistic pathogen, *P. aeruginosa* is considered to be a leading cause of nosocomial infections among patients with immunocompromised states, cystic fibrosis (CF), burn injuries, as well as urinary tract infections, pneumonia, and bacteremia^3^. Hospital-acquired infections caused by *P. aeruginosa* account for about 10% of all nosocomial infections, and in more serious cases the mortality rate is as high as 50%^1,3^. The efficacy of conventional antibiotics therapy for PA infections is always limited due to intrinsic and acquired mechanisms of drug resistance, including active efflux, target mutations, the expression of antibiotic-inactivating enzymes, and biofilm formation^4^. Despite new antibiotic approvals, resistance in PA is rising faster, doubling in the past three years^5^. Carbapenem-resistant *P. aeruginosa* (CRPA) is considered “critical-priority” bacterium among ESKAPE pathogens by the world health organization (WHO) since 2017^4,6^. Hence, the development of effective vaccines and alternative immunotherapies is urgently warranted against PA infection and epidemics^7^.

In the past half-century, extensive research has been focused on the development of anti-*P. aeruginosa* vaccines. However, no vaccine has been approved for clinical use^7^. Several important antigens, including lipopolysaccharide (LPS), alginate, flagellum, outer membrane proteins (OMPs), type 3 secretion systems (T3SSs), and killed whole-cell have been targeted in clinical development^8^. Nevertheless, to date, only three vaccine candidates have reached the phase III clinical trials: an octavalent O-polysaccharide-exotoxin A conjugate (Aerugen®)^9^, a bivalent flagella vaccine^10^, and His-tagged outer membrane protein OprF-OprI fusion protein (IC43)^8,11^. Among them, the protein subunit vaccine IC43 seems to be the most effective candidate due to the better safety and immunogenicity profile of the phase II study. The overall mortality rate in IC43-vaccinated patients did not appear to be statistically different from the placebo group.

T3SS is one of the most important virulence factors of PA, which injects bacterial toxins into host cells through a needle-like complex, facilitating immune escape and enabling bacterial colonization^12^. The *P. aeruginosa* V-antigen (PcrV) is a critical needle-tip protein of T3SS which is indispensable for forming pores on the host cell membrane to translocate effector toxins into host cells^12,13^. PcrV is highly conserved^14^ and well-validated to be a good target for immunoprophylactic strategies against *P. aeruginosa* in animal models^15^. Indeed, immunization with PcrV DNA vaccine could elicit protective immunity against acute pneumonia and decrease lung inflammation^16,17^. Recent research demonstrated that nasal vaccination with PcrV adjuvanted with CpG was capable to induce PcrV-specific IgG and IgA antibodies and protect from *P. aeruginosa*-caused pneumonia^18^. Besides, anti-PcrV antibodies are evidenced to combat PA infection in animal models^19^ and even in CF patients. The phase II clinical trial of anti-PcrV Fab fragment (KB001) demonstrated that it could reduce inflammation and damage of the airways in CF patients^20^. Results of phase I clinical trial with healthy subjects support further evaluation of the safety and efficacy of bispecific antibodies targeting PcrV and Psl in subjects with possible PA pneumonia^21^. Moreover, polyclonal anti-PcrV IgGs isolated from human serum were found to confer protection against *P. aeruginosa*-caused pneumonia in mice^22,23^. A phase 2, randomized, double-blind, parallel-group, placebo-controlled study (NCT02696902) showed that the bivalent, bispecific human immunoglobulin G1 kappa monoclonal antibody MEDI3902 which can selectively bind to the PA PcrV and Psl, could not reduce PA nosocomial pneumonia incidence in PA-colonized mechanically ventilated patients^24^. The abovementioned suggests that PcrV is a promising antigen for PA vaccines compared with a number of other candidate antigens.

Membrane porin F (OprF) and lipoprotein I (OprI) are the most widely studied outer membrane proteins as target antigens identified in *P. aeruginosa*^25,26^ and are highly conserved and immunogenic across all *P. aeruginosa* strains^27,28^. Indeed, protein subunit vaccine IC43 that has entered into the phase III clinical trial used OprF (190-342) and OprI (21-83) as antigens of recombinant protein^11,29^. Although the clinical trial (NCT01563263) indicated that IC43 immunization provided no clinical benefit for vaccinated patients with mechanical ventilation in the intensive care unit, preclinical investigation and earlier phase clinical study have demonstrated good immunogenicity and safety^11^.

The coronavirus disease 2019 (COVID-19) pandemic has boosted the most unprecedented vaccine development in history, with mRNA vaccines progressing from concept to clinical reality^30^. mRNA vaccines are a better alternative to traditional vaccine approaches because of their low-cost manufacturing advantages, the potential for safe administration, and most importantly, their ability to be efficiently and rapidly developed^31–33^. In the current study, we selected PcrV and OprF-I as antigens, designed two mRNA vaccine constructs, and evaluated the immune responses as well as the protection capacity of the proposed anti-PA mRNA vaccine candidates as well as PcrV and OprF-I protein vaccine candidates.

## Results

### mRNA-PcrV-LNP and mRNA-OprF-I-LNP preparation and in vitro characterization

**Fig. 1:**
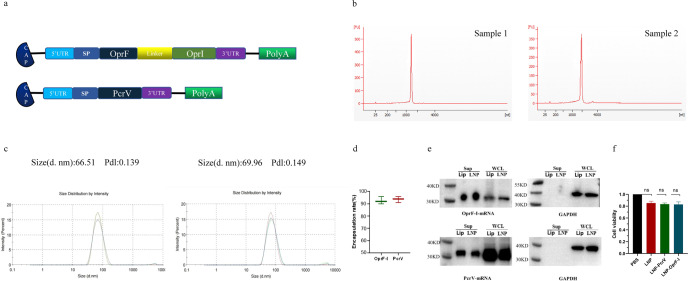
mRNA-PcrV and mRNA-OprF-I vaccine design and in vitro characterization. **a** The schematic illustration of mRNA-PcrV and mRNA-OprF-I constructs. The mRNA constructs consist of 5’ cap followed by 5’UTR, signal peptide, OprF, linker, OprI, 3’UTR, and polyA tail. **b** The capillary electrophoresis profiles of in vitro synthesized PcrV mRNA and OprF-I mRNA. Sample 1 denotes the mRNA-PcrV, while sample 2 denotes mRNA-OprF-I. The electropherogram shows the optimal purity of synthesized mRNAs. **c** The size distribution of LNPs was measured by a Malvern particle size instrument. **d** The encapsulation efficiency of LNPs determined by the Ribogreen assay. The figure indicates the optimal encapsulation of mRNA-PcrV and mRNA-OprF-I. **e** The expression and secretion of mRNA vaccines 48 h after transfection into HEK-293T cells detected by WB. It is shown that both mRNAs were successfully expressed in cells as well as secreted into the supernatants. **f** The cytotoxicity of blank LNP and mRNA vaccines in HEK293 cells. 5’UTR, 5’ untranslated region, SP signal peptide, OprF membrane porin F, OprI lipoprotein I, 3’UTR 3’ untranslated region, PolyA polyadenylic acid tail, PcrV *P. aeruginosa* V-antigen, PDI Polydispersity Index, Lip lipofectamine 2000, Sup supernatant, WCL whole cell lysate. Data are presented as means ± SEM.

To develop broad and effective vaccine candidates against *P. aeruginosa*, we designed two mRNA vaccines encoding the full-length of PcrV and a fusion protein of outer membrane proteins OprF (amino acid residues 190–342) and OprI (amino acid residues 21–83), respectively (Fig. 1a). Both proteins contained a C-terminal 6xHis tag for protein expression detection. mRNA vaccines were synthesized via T7 polymerase-mediated in vitro transcription with N1-methyl-pseudouridine nucleoside substituting of uridine (U). The synthesized mRNA molecules were shown to be of high integrity as determined by capillary electrophoresis (Fig. 1b). The open reading frame (ORF) of both mRNA constructs are given in supplementary Note 1. mRNA was encapsulated into lipid nanoparticle (LNP) via a microfluidic system for in vivo delivery, and the average size of resulting particles detected by dynamic light-scattering was 66.51 nm and 69.96 nm, respectively (Fig. 1c). The encapsulation efficiency of both mRNA-LNP vaccines was higher than 95% as detected by Quant-iT ™ RiboGreen ™ RNA kit (Fig. 1d). Furthermore, transfection of HEK293T cells with mRNA- PcrV-LNP and mRNA-OprF-I-LNP achieved a high level of target proteins’ expression and secretion into cell medium due to the introduction of secretion signal peptide (Fig. 1e). Besides, low cytotoxicity was detected (Fig. 1f). Cryo-TEM image of LNP wrapped mRNA is given in Supplementary Figure 1. The original blots are given in Supplementary Figure 2.

### mRNA-PcrV-LNP and mRNA-OprF-I-LNP immunization elicited strong antigen-specific humoral and T-cell immune responses in BALB/c mice

**Fig. 2:**
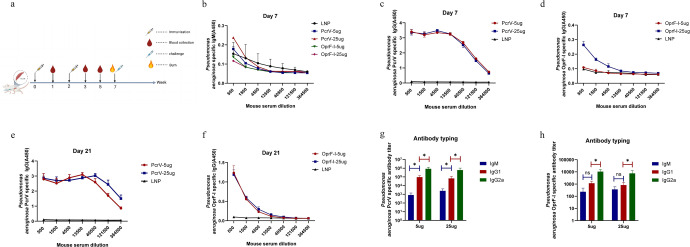
Humoral immune response of BLAB/c mice activated by the immunization with mRNA-PcrV and mRNA-OprF-I vaccines. **a** Timeline of mice immunization, blood collection and PA challenging. **b** The IgM levels induced by mRNA vaccines seven days after the first immunization with different doses of mRNA-PcrV-LNP and mRNA-OprF-I-LNP. **c** Specific IgG titers in mice sera seven days after the first immunization with mRNA-PcrV-LNP. **d** Specific IgG titers in mice sera seven days after the first immunization with mRNA-OprF-I-LNP. **e** Specific IgG titers in mice sera seven days after the second immunization with mRNA-PcrV-LNP. **f** Specific IgG titers in mice sera seven days after the second immunization with mRNA-OprF-I-LNP. **g** IgM, IgG1, and IgG2a titers in mRNA-PcrV-LNP immunized mice sera. **h** IgM, IgG1, and IgG2a titers in mRNA-OprF-I-LNP immunized mice sera. Mice in each group *n* = 8. One-way ANOVA was performed (ns non-significance, *P < 0.05, **P < 0.01, ***P < 0.001). Data are presented as means ± SEM.

To determine the immune response induced by PcrV and OprF-I mRNA vaccines, groups of BALB/c mice (*n* = 8) were immunized intramuscularly twice with high (25 µg) or low (5 µg) dose of mRNA vaccines with empty LNP immunization as control. The interval between every injection was two weeks (Fig. 2a). The blood samples were collected at the indicated time and antigen-specific antibodies were detected by ELISA. The ELISA plates were coated with recombinant, *E.coli*-expressed, and purified PcrV or OprF-I (Supplementary Figure 3). IgM generation after seven days of immunization was not significantly different from the control group (Fig. 2b). As shown in Fig. 2c and Fig. 2d the OprF-I specific IgG titer was slightly increased after initial immunization, while dramatically increased after the second immunization. Moreover, the IgG titer in serum samples of mice immunized with the PcrV-mRNA vaccine was significantly higher than that in mice immunized with OprF-I at all time points examined (Fig. 2e and Fig. 2f), and immunization with 5 µg of PcrV mRNA vaccine could induce almost the same level of IgG titer as 25 µg induced (Fig. 2e and Fig. 2f). Encouragedly, the level of IgG was rapidly increased in mice that received a single immunization of PcrV mRNA vaccine (7 days post priming) and reached a level equivalent of booster vaccination, suggesting that this vaccine could provide protection against PA infection quickly after a single vaccination.

**Fig. 3:**
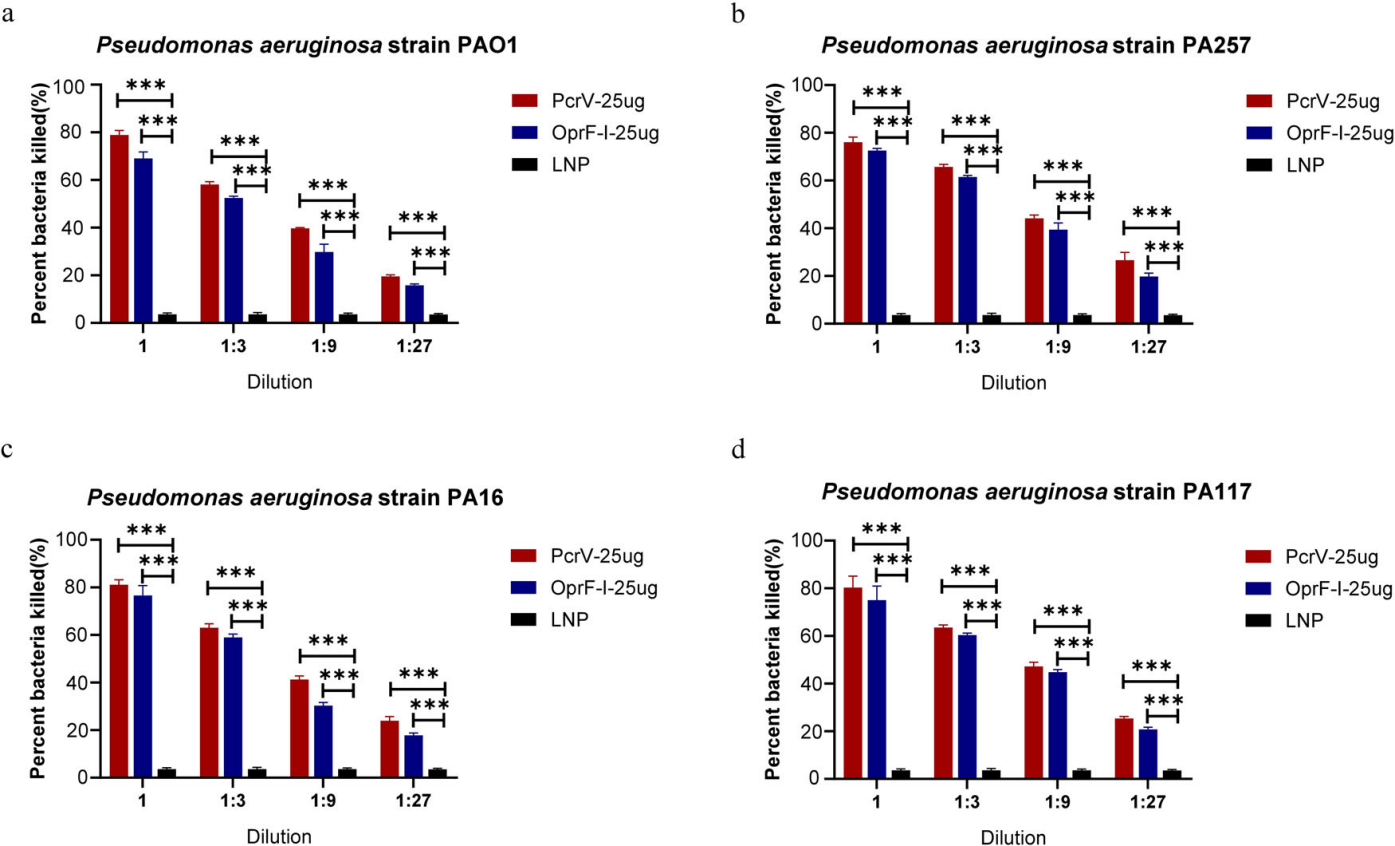
Opsonophagocytic killing (OPK) activity in the sera of mice immunized with mRNA-PcrV-LNP and mRNA-OprF-I-LNP. **a** OPK activity against PAO1 strain. **b** OPK activity against PA257 strain. **c** OPK activity against PA16 strain. **d** OPK activity against PA117 strain. Mice in each group *n* = 8. Two-tailed Student’s t-test was performed (ns non-significance, *P < 0.05, **P < 0.01, ***P < 0.001). Data are presented as means ± SEM.

**Fig. 4:**
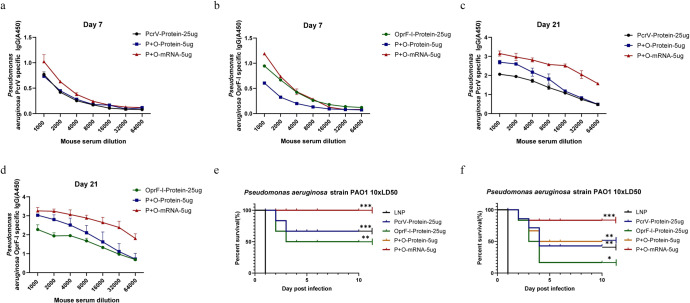
Humoral immune response of BLAB/c mice induced by the immunization. **a** Specific IgG titers in mouse serum seven days after the first immunization with PcrV protein, combination of PcrV + OprF-I protein, and combination of mRNA-PcrV + mRNA-OprF-I. **b** Specific IgG titers in mouse serum seven days after the first immunization with OprF-I protein, combination of PcrV + OprF-I protein, and combination of mRNA-PcrV + mRNA-OprF-I. **c** Specific IgG titers in mouse serum twenty-one days after the first immunization with PcrV protein, combination of PcrV + OprF-I protein, and combination of mRNA-PcrV + mRNA-OprF-I. **d** Specific IgG titers in mouse serum seven days after the first immunization with OprF-I protein, combination of PcrV + OprF-I protein, and combination of mRNA-PcrV + mRNA-OprF-I. **e** The survival rate of mice protected by PcrV protein, OprF-I protein, mixture of PcrV and OprF-I proteins, and a mixture of mRNA-PcrV + mRNA-OprF-I vaccines in a challenge experiment with 10xLD_50_ PAO1 strains in a burn model. On the graph blue indicates the overlap of PcrV-protein-25µg (blue) and P + O-Protein-µg (yellow) curves. **f** The survival rate of mice protected by PcrV protein, OprF-I protein, mixture of PcrV and OprF-I proteins, and a mixture of mRNA-PcrV + mRNA-OprF-I vaccines in a challenge experiment with 10xLD_50_ PAO1 strains in a systemic infection model. Mice in each group *n* = 8. Data are presented as means ± SEM. The data for survival test were analyzed by Wilcoxon log-rank survival test (ns non-significance, *P < 0.05, **P < 0.01, ***P < 0.001).

In general, Th1 immune responses mainly induce IgG2a antibody production, while significant changes in IgG1 are mainly caused by Th2 immune responses. In this study, total IgG, IgG1, and IgG2a were measured by ELISA to characterize antigen-directed humoral immune responses (Th1 vs. Th2-response) induced by mRNA-PcrV-LNP and mRNA-OprF-I-LNP immunization. As shown in Fig. 3f and Fig. 3g, both IgG1 and IgG2a were abundantly present in the sera of mice immunized with both antigens, but the level of IgG2a was a little higher than IgG1 (Fig. 2g), indicating that immunization with both vaccines elicited a Th1/Th2 mixed or slightly Th1-biased immune response. Except for evaluating the immune response elicited by mRNA-PcrV-LNP and mRNA-OprF-I-LNP immunization, the mice were also vaccinated with the mixed mRNA-PcrV-LNP and mRNA-OprF-I-LNP vaccine. Compared with the protein vaccines, combination of mRNA vaccine showed the most abundant production of IgG in sera (Fig. 4a-d).

To further explore whether the immunized mice sera could protect cells against PA infection, we performed an opsonophagocytic killing (OPK) assay to determine the activity of the induced antibodies against bacterial killing under complement-mediated conditions according to reported methods. As expected, bacterial killing activity was not observed from blank LNP immune sera and mice sera from 25 µg PcrV or 25 µg OprF-I –vaccinated mice demonstrated good bacterial killing activities under complement-mediated conditions, and the activity was observed in a concentration-dependent manner (Fig. 3). Moreover, the phagocytic and bactericidal activity of PcrV-immunized mice sera was little higher than that of OprF-I-immunized against all the four strains tested (Fig. 3a–d).

**Fig. 5:**
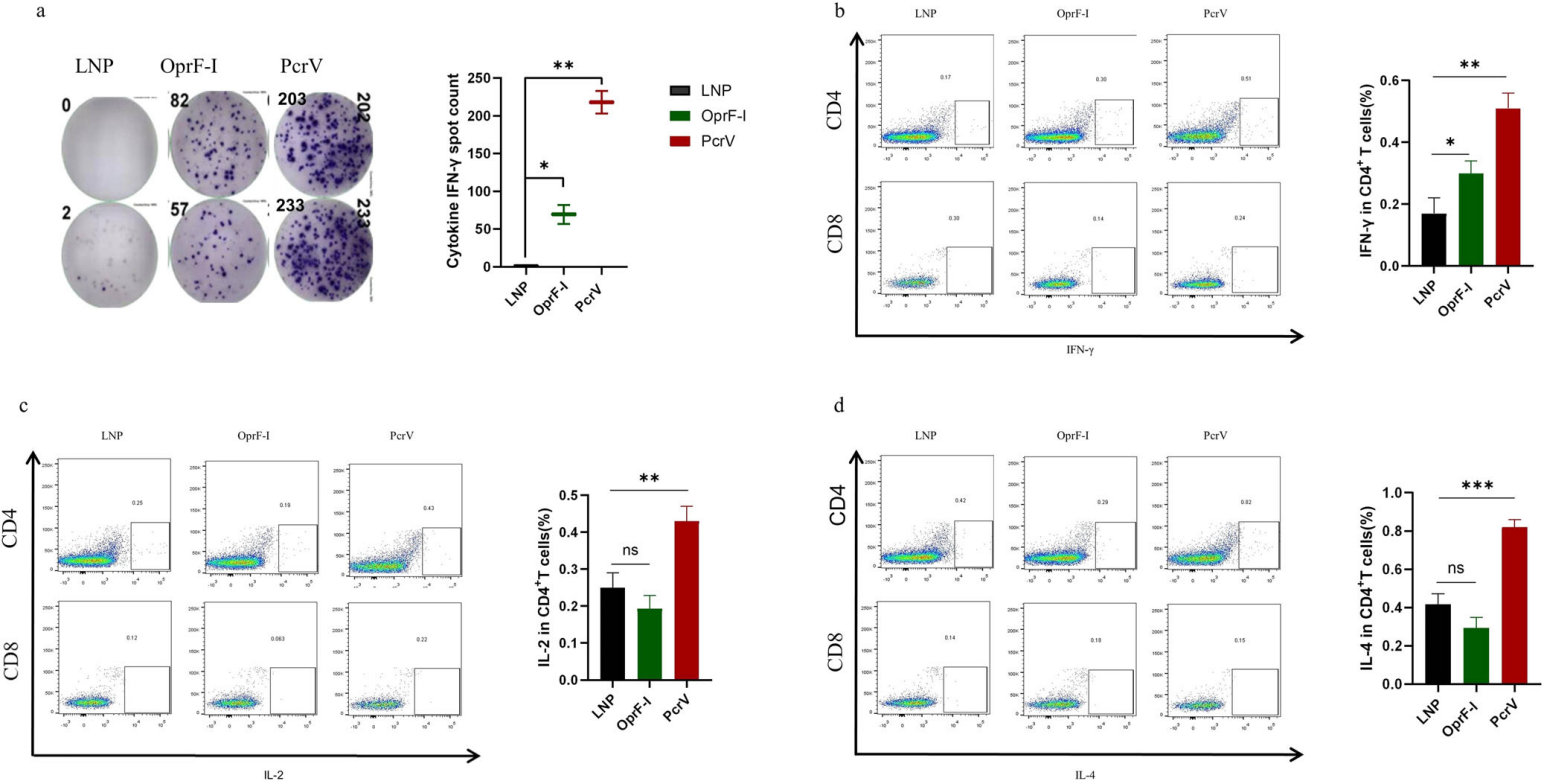
Cellular immune responses in BALB/c mice immunized with mRNA vaccine. **a** Image of the Elispot assay plate and the spot counts of IFNγ-producing T cells detected by Elispot. **b** The percentages of IFNγ-producing CD4+ and CD8 + T cells detected by intracellular cytokine staining. **c** The percentages of IL-2-producing CD4+ and CD8 + T cells detected by intracellular cytokine staining. **d** The percentages of IL-4-producing CD4+ and CD8 + T cells detected by intracellular cytokine staining. Mice in each group *n* = 8. One-way ANOVA was performed (ns non-significance, *P < 0.05, **P < 0.01, ***P < 0.001). Data are presented as means ± SEM.

In addition to antibody production, we further evaluated cellular immune responses induced by intramuscular vaccination of BALB/c mice with mRNA-PcrV-LNP or mRNA-OprF-I-LNP two weeks after the second immunization. Mice immunized with an empty LNP served as negative controls. Remarkably, the enzyme-linked immunospot (ELISpot) assay indicated that the splenocytes obtained from mice immunized with mRNA-PcrV-LNP exhibited much higher frequencies of IFN-γ-secreting cells than mice immunized with mRNA-OprF-I-LNP after stimulation with appropriate peptide mixtures. The peptides were selected from the Immune Epitope Database (IEDB) or predicted via IEDB analysis resources (the sequences of peptides are listed in Supplementary Table 1). The frequencies of IFN-γ-secreting cells in the spleens were at background levels when there was no stimulation (Fig. 5a). In addition, the flow cytometry demonstrated that the secretion of IFN-γ (Fig. 5b), IL2 (Fig. 5c), and IL4 (Fig. 5d) (Supplementary Figure 4) in mRNA-PcrV-LNP immunized group was significantly increased in CD4^+^ cells compared with CD8^+^ cells after peptides simulation, while no obvious change in OprF-I-mRNA-LNP immunized group was observed suggesting that intramuscular vaccination with mRNA-PcrV-LNP mainly induces CD4^+^ T cell responses.

### Vaccination with mRNA-PcrV-LNP and mRNA-OprF-I-LNP protects against *Pseudomonas aeruginosa* infections in both burn wound and systemic infection models

**Fig. 6:**
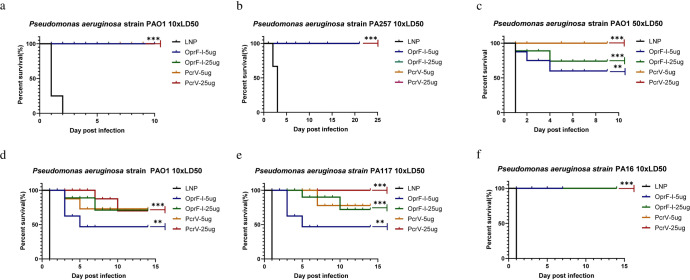
Protective effect of mRNA-PcrV-LNP and mRNA-OprF-I-LNP vaccines in mice. **a** The survival rate of mice protected by mRNA-PcrV-LNP and mRNA-PcrV-LNP vaccines after a challenging with 10xLD_50_ PAO1 strain in a burn model. **b** The survival rate of mice protected by mRNA-PcrV-LNP and mRNA-PcrV-LNP vaccines after a challenging with 10xLD_50_ PA257 strain in a burn model. **c** The survival rate of mice protected by mRNA-PcrV-LNP and mRNA-PcrV-LNP vaccines after a challenging with 50xLD_50_ PAO1 strains in a burn model. **d** The survival rate of mice protected by mRNA-PcrV-LNP and mRNA-PcrV-LNP vaccines after a challenging with 10xLD_50_ PAO1 strain in a systemic infection model. **e** The survival rate of mice protected by mRNA-PcrV-LNP and mRNA-PcrV-LNP vaccines after a challenging with 10xLD_50_ PA16 strain in a systemic infection model. **f** The survival rate of mice protected by mRNA-PcrV-LNP and mRNA-PcrV-LNP vaccines after a challenging with 10xLD_50_ PA117 strain in a systemic infection model. Mice in each group *n* = 8. Data are presented as means ± SEM. The data for survival test were analyzed by Wilcoxon log-rank survival test (ns non-significance, *P < 0.05, **P < 0.01, ***P < 0.001).

To determine whether mRNA vaccination could provide broad protection, immunized mice were challenged with standard PA strain PAO1 (a wild-type serogroup O2/O5 strain) and three other PA clinical strains PA 257, PA16, and PA 117 isolated from hospitals in China at the doses of 10×LD_50_, or 50×LD_50_ four weeks after the second injection, and the survival was monitored for two weeks. The results of exotoxin analysis of the selected PA strains, median lethal doses of PAO1 and PA257 in burn models and PAO1, PA16, and PA117 in mouse systemic infection models, as well as antibiotic resistance of the selected PA strains and the clinical background of PA isolates are given in Supplementary Figure 5, Supplementary Table 2, 3, 4, and 5, respectively. The four strains show different levels of virulence factor ExoU (the most virulent T3SS effector) and drug resistance ability. Based on the data, PA strains could be ranked as PA257 > PA117 > PA16 > PAO1 according to the ExoU production (Supplementary Figure 5) and as PA117 > PA257 > PA116 > PAO1 according to the multidrug resistance (Supplementary Table 4). Hence, PA 257 and PA117 strains were selected to use in animal models. Besides, since PAO1 is a standard strain, it was also included in all the experiments. As shown in Fig. 6, all the mice in the control group died within 2 days after infection. In contrast, all the mice in PcrV or OprF-I immunized groups survived when challenged with 10×LD_50_ of PAO1 or PA257 in burned infection model (Fig. 6a, b). To further differentiate the protective effect of the two vaccines, we increased the challenging dosage of PA strain PAO1 to 50×LD_50_. As demonstrated in Fig. 6c, the survival rate of PcrV-immunized groups (5 µg and 25 µg) kept as high as 100%, while the survival rate of the 25 µg OprF-I-immunized group dropped to 75%, and the survival rate of the 5 µg OprF-I-immunized group dropped to 50% in a burned infection model.

The survival rate of burn mouse and systemic infection models immunized with the combination of mRNA-PcrV + mRNA-OprF-I were 100% and 85%, respectively, after challenging with 10×LD50 of PAO1 strain. In the systemic infection model, the survival rates of 5 µg mRNA-OprF-I, 25 µg mRNA-OprF-I, 5 µg mRNA-PcrV, and 25ug mRNA-PcrV immunized mice were 50%, 75%, 75%, and 75%, respectively (Fig. 6d), when challenged with 10×LD50 of PAO1. The survival rates of the above four groups were 50%, 62.5%, 75%, and 100%, respectively, when challenged with 10×LD50 of PA117 (Fig. 6f). Unlike the challenged groups with PA strains PAO1 and PA117, no death was observed in the above four groups of mRNA vaccine-immunized mice when challenged with 10×LD50 of PA16 (Fig. 6e). In case of recombinant protein-vaccinated mice, the best protection in both burned (almost 70%) and systemic infection models (~50%) was observed in mice immunized with the combined vaccine containing both, PcrV and OprF-I proteins and PcrV, respectively. The mRNA combined vaccine containing both mRNA-PcrV-LNP and mRNA-OprF-I-LNP showed the superior protection over protein vaccines after challenging with 10×LD50 of PAO1 strain in both burn and systemic infection models (Fig. 4e-f). Taken together, these data suggest that a PcrV vaccine may provide better protection against multiple strains of PA. Additionally, immunization with mRNA vaccine has better outcome compared with the protein vaccine.

### Vaccination with PcrV and OprF-I vaccines protects mice from infection by reducing bacterial burden and inflammation

**Fig. 7:**
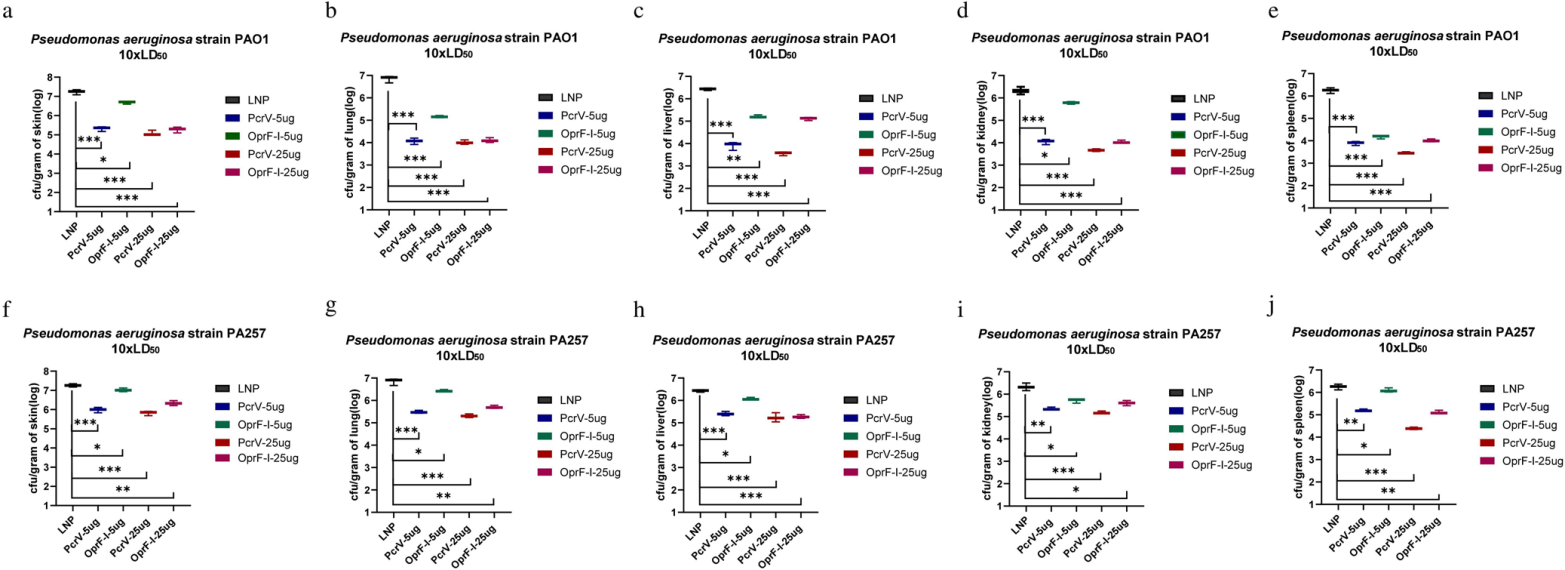
Bacterial colonization of organs following the challenge in mRNA-immunized mouse burn models. **a** Bacterial counts per gram of mice skin after challenge with standard strain PAO1 10xLD_50_ in burn model. **b** Bacterial counts per gram of mice lung after challenge with standard strain PAO1 10xLD_50_ in burn model. **c** Bacterial counts per gram of mice liver after challenge with standard strain PAO1 10xLD_50_ in burn model. **d** Bacterial counts per gram of mice kidney after challenge with standard strain PAO1 10xLD_50_ in burn model. **e** Bacterial counts per gram of mice spleen after challenge with standard strain PAO1 10xLD_50_ in burn model. **f** Bacterial counts per gram of mouse skin after challenge with clinical strain PA257 10xLD_50_ in a burn model. **g** Bacterial counts per gram of mouse lung after challenge with clinical strain PA257 10xLD_50_ in a burn model. **h** Bacterial counts per gram of mouse liver after challenge with clinical strain PA257 10xLD_50_ in a burn model. **i** Bacterial counts per gram of mouse kidney. after challenge with clinical strain PA257 10xLD_50_ in a burn model. **j** Bacterial counts per gram of mouse spleenafter challenge with clinical strain PA257 10xLD_50_ in a burn model. Mice in each group *n* = 8. One-way ANOVA was performed (ns non-significance, *P < 0.05, **P < 0.01, ***P < 0.001). Data are presented as means ± SEM.

**Fig. 8:**
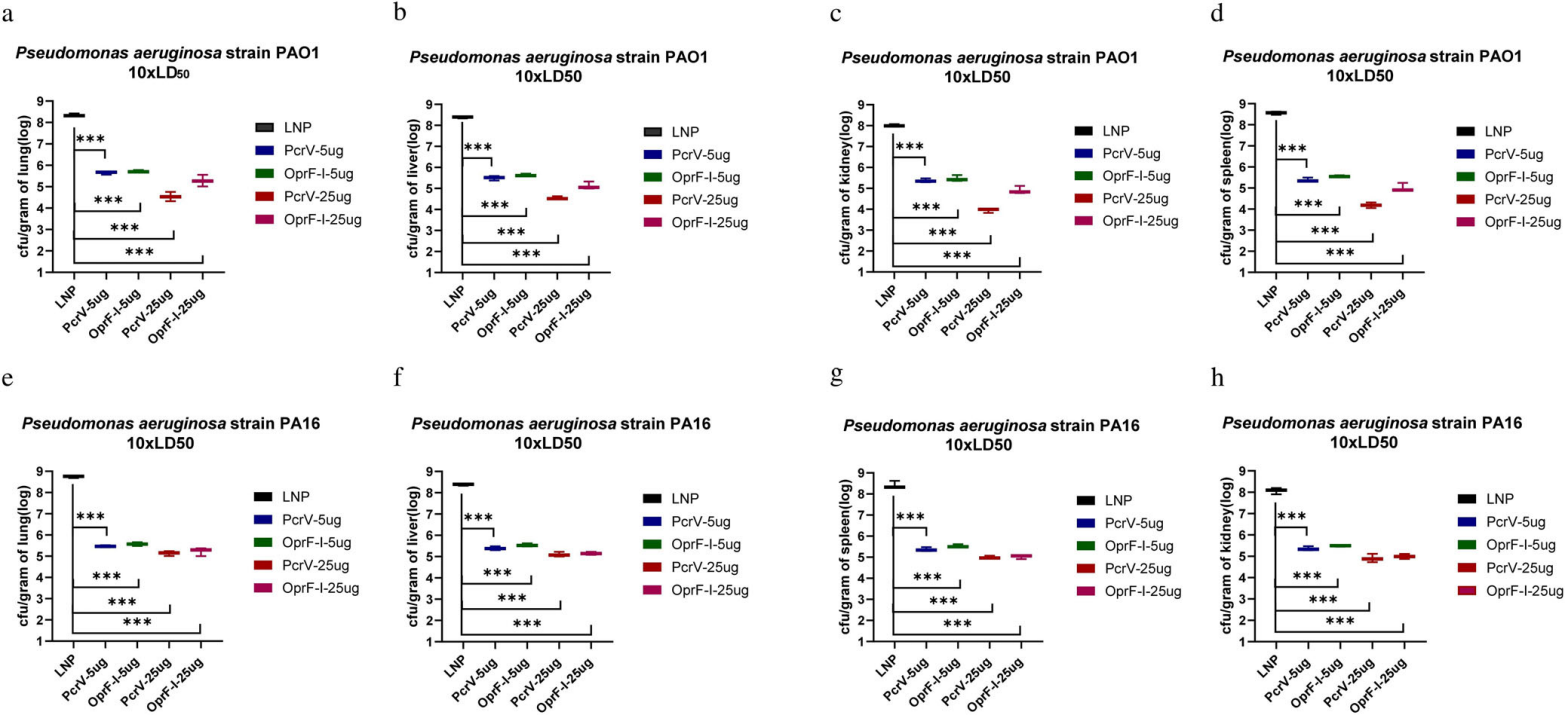
Bacterial colonization of organs after challenge in mRNA-immunized mouse systemic infection models. Bacterial counts per gram of lung (**a**), liver (**b**), spleen (**c**) and kidney (**d**) after challenge with standard strain PAO1 10xLD_50_ in systemic infection model. Bacterial counts per gram of lung (**e**), liver (**f**), spleen (**g**) and kidney (**h**) after challenge with clinical strain PA16 10xLD_50_ in mouse systemic infection model. Mice in each group *n* = 8. One-way ANOVA was performed (ns non-significance, *P < 0.05, **P < 0.01, ***P < 0.001). Data are presented as means ± SEM.

**Fig. 9:**
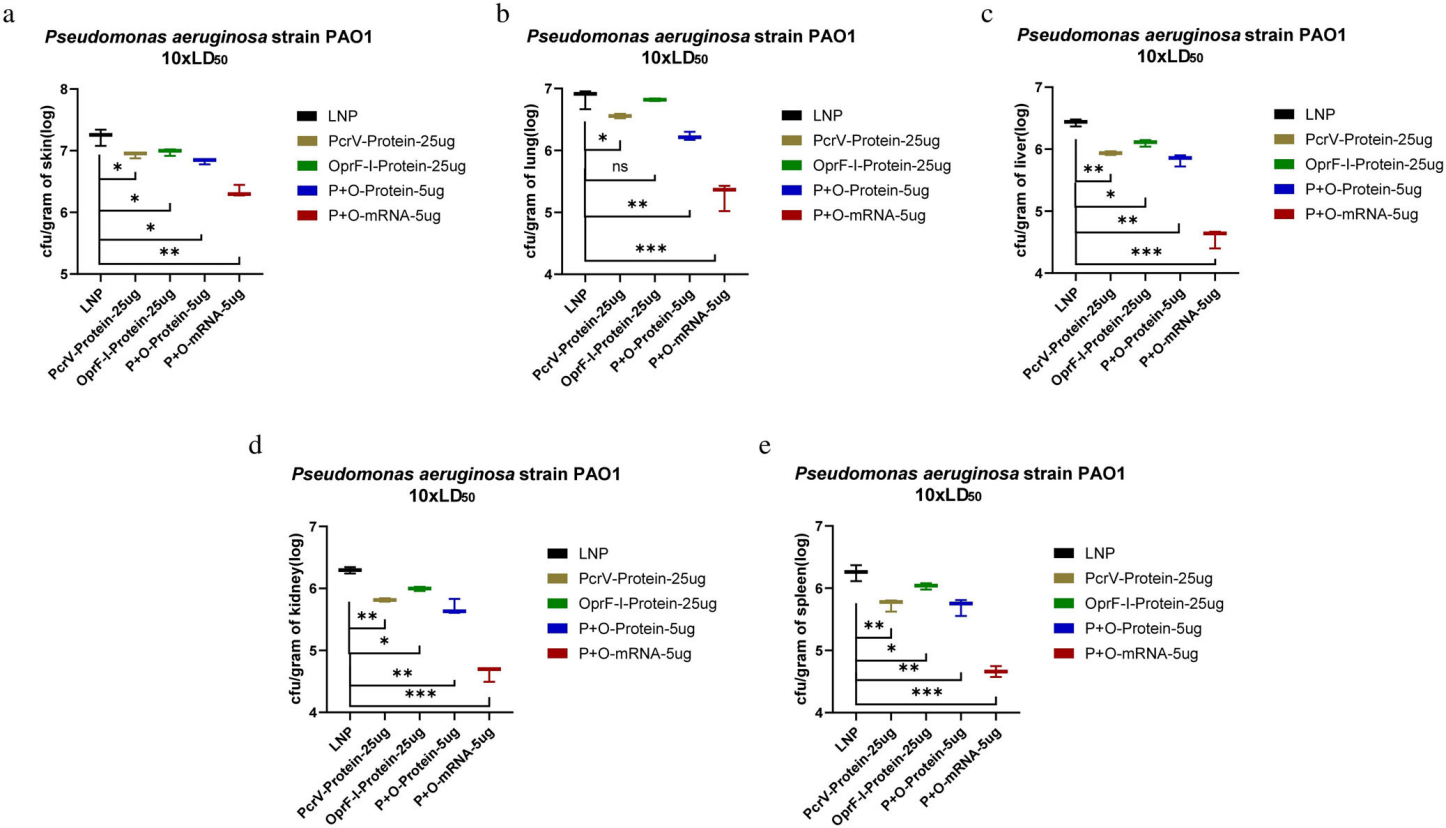
Bacterial colonization of organs in protein or mixed mRNA vaccine-immunized burn models after the challenge with standard strain PAO1 10xLD_50_. **a** Bacterial counts per gram of mouse skin. **b** Bacterial counts per gram of mouse lung. **c** Bacterial counts per gram of mouse liver. **d** Bacterial counts per gram of mouse kidney. **e** Bacterial counts per gram of mouse spleen. Mice in each group *n* = 8. One-way ANOVA was performed (ns non-significance, *P < 0.05, **P < 0.01, ***P < 0.001). Data are presented as means ± SEM.

**Fig. 10:**
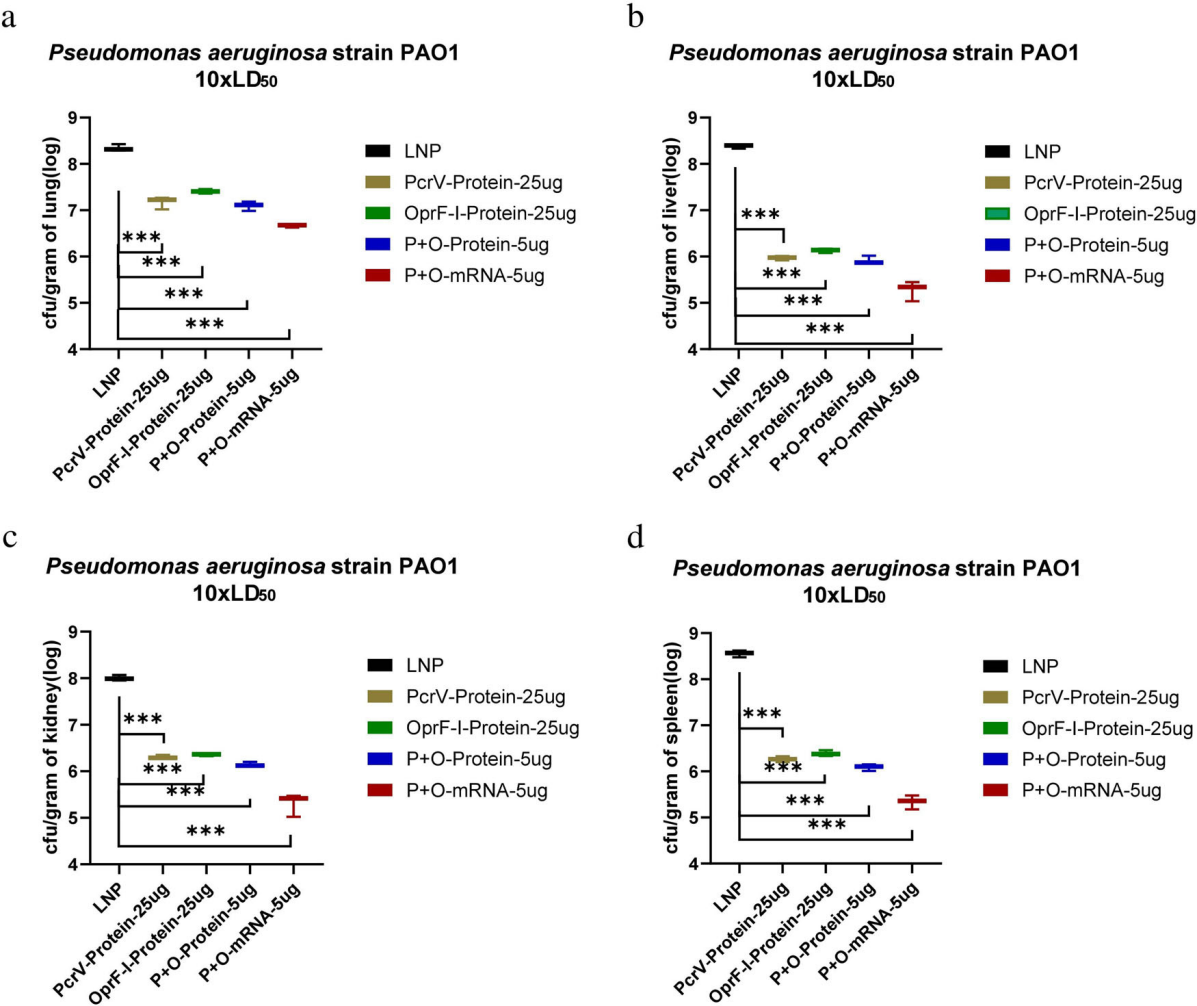
Bacterial colonization of organs in protein or mixed mRNA vaccine-immunized mouse systemic infection models after the challenge with standard strain PAO1 10xLD_50_. **a** Bacterial counts per gram of mouse lung. **b** Bacterial counts per gram of mouse liver. **c** Bacterial counts per gram of mouse kidney. **d** Bacterial counts per gram of mouse spleen. Mice in each group *n* = 8. One-way ANOVA was performed (ns non-significance, *P < 0.05, **P < 0.01, ***P < 0.001). Data are presented as means ± SEM.

Twenty-four hours after the bacterial challenge, the organs were collected and bacterial colonization was determined in the lung, liver, spleen, and kidney. Analyses of bacterial colonization of organs 24 h post-infection in immunized mouse burn models demonstrated that immunization with both, PcrV and OprF-I mRNA vaccines significantly lowered bacterial loads compared with the control group, both in case of challenge with PA strains PAO1 (Fig. 7a-e) and PA257 (Fig. 7f-j). Similarly, analyses of bacterial burden in organs following the challenge in immunized mouse models of systemic infection showed significantly lower bacterial loads in each organ after the challenge with PA strains PAO1 (Fig. 8a-d), PA 16 (Fig. 8e-h), and PA 117 (Supplementary Figure 6). Moreover, the colonization was much lower in organs of mRNA-PcrV-immunized mice compared with mRNA-OprF-I-immunized mice. These analyses demonstrated that immunization with mRNA-PcrV-LNP or mRNA-OprF-I-LNP could simultaneously reduce bacterial colonization and systemic transmission in organs, and the effect of mRNA-PcrV-LNP immunization was superior to mRNA-OprF-I-LNP (Fig. 7a-c and Fig. 8a-c). In case of recombinant-protein vaccinated mice, the mix-protein (PcrV+OprF-I) demonstrated lower colonization after infection with the PAO1 strain compared to the separate protein-vaccinated mice. Additionally, the lowest colonization was observed in tissues of mice that were vaccinated with the mix of mRNA vaccines– mRNA-PcrV-LNP and mRNA-OprF-I-LNP (Fig. 9 and Fig. 10).

**Fig. 11:**
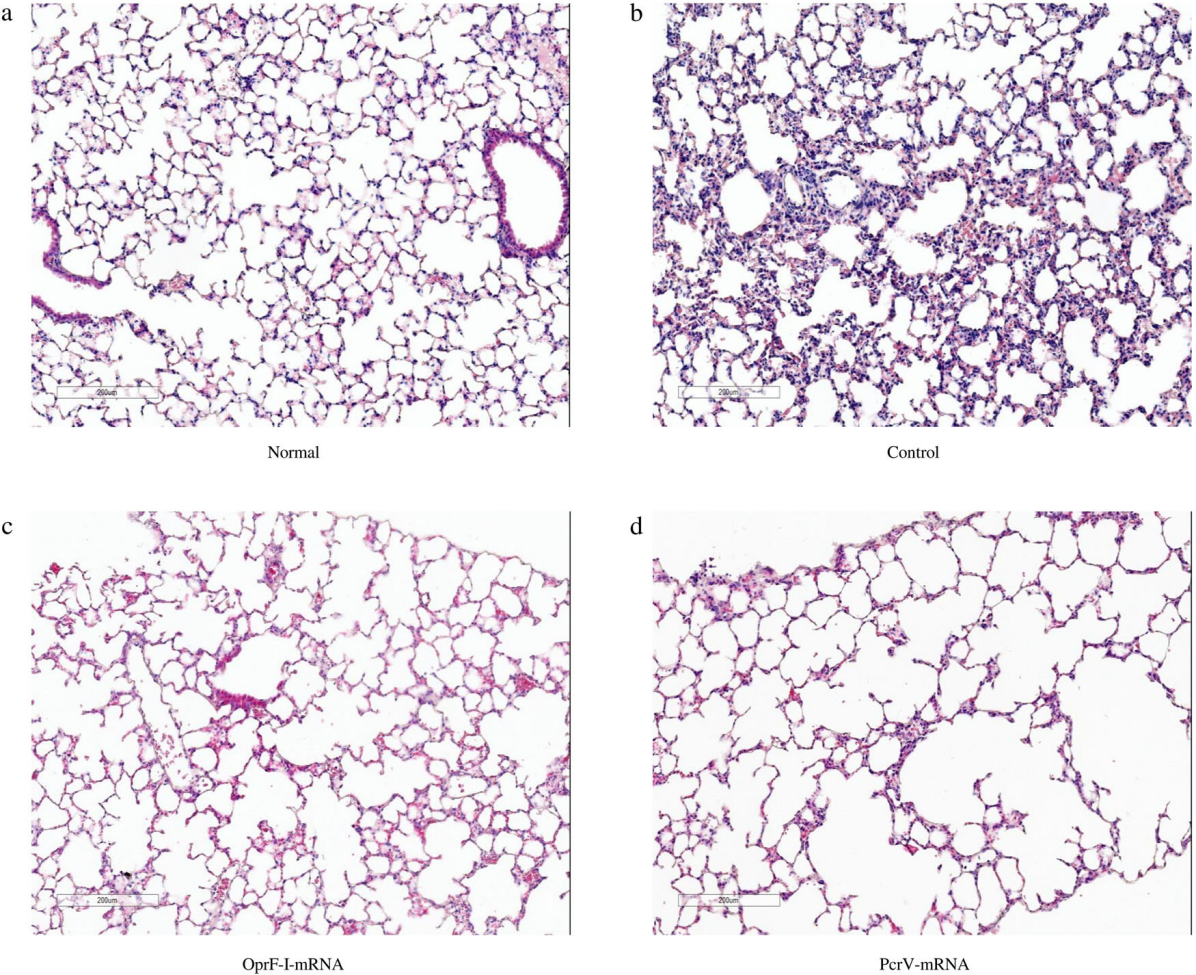
Protective effect of mRNA vaccines on the lungs of immunized mice. **a** HE staining image of normal mouse lung pathological sections. **b** HE staining image of control mouse lung pathological sections. **c** HE staining image of mRNA-OprF-I-LNP-immunized mouse lung pathological sections. **d** HE staining image of mRNA-PcrV-LNP-immunized mouse lung pathological sections. HE Hematoxylin and eosin. Bars = 200μm.

Histological analysis of the lungs in burned infection model that were collected twenty-four hours after challenging showed that neither inflammation nor injury was detected in the uninfected LNP group (Fig. 11a) while enhanced neutrophil infiltration, alveolar hemorrhage, and destruction of the alveolar structure were observed in the blank-LNP immunized mice (Fig. 11b). In contrast, immunization with mRNA-PcrV-LNP (Fig. 11c) and mRNA-OprF-I-LNP (Fig. 11d) substantially reduced the inflammatory changes and tissue damage, suggesting PcrV and OprF-I vaccination in advance can significantly reduce lung injury caused by PA infection.

## Discussion

Despite the availability and tremendously successful application of conventional vaccines, there are still many infectious pathogens against which no efficacious vaccines are accessible, PA, which can cause life-threatening health complications is among them^34^. PA is commonly found in aquatic environments such as fresh water and soil^35^. As an opportunistic pathogen PA can infect immunocompromised hosts and raise a global concern. It worsens the inflammatory status and pulmonary function when colonizing CF lungs^35^. Being able to form biofilms on implanted devices, PA often causes infections that are difficult to detect and treat with antibiotic therapy^36^ as the resistance to the antibiotics through the acquired or intrinsic mechanisms makes the treatment challenging. The resistance is transferred via interchangeable genetic materials that lays the groundwork for the prevalence of antibiotic-resistant strains^37^. After a retrospective study of the development history of PA vaccines, we find all the developing vaccines only adopt traditional vaccine technologies, including inactivated, subunit, and conjugated vaccines which is possibly the reason that has limited the success of PA vaccines^38^. All the above-mentioned indicates the necessity of developing effective vaccine candidates against this emerging pathogen. Remarkably, nucleic acid vaccines demonstrate extremely favorable outcomes in terms of time and cost-effectiveness, safety, and efficacy^33,39–43^. Indeed, nucleic acid vaccines have shown high efficiency against the ongoing COVID-19 pandemic, particularly, two mRNA vaccines against severe acute respiratory syndrome coronavirus 2 (SARS‑CoV‑2) (BNT162b2 by BioNTech/Pfizer and mRNA-1273 by Moderna) authorized by WHO for an emergency use^39^ and a DNA vaccine for SARS-CoV-2 approved in India^44^. Remarkably, among the nucleic acid vaccines, mRNA technology elicits prominent features. Accordingly, developing an mRNA-based vaccine against PA indeed makes sense. Currently, mRNA technology as a novel vaccine approach is mostly utilized in the field of viral infections and cancer immunotherapy and is rarely used for bacterial infections^45^. In this study, we built an mRNA vaccine platform for the development of a vaccine candidate against PA, hoping to resolve the unmet need for PA infection prevention.

One of the greatest challenges for the development of PA vaccines is the selection of antigen targets. To date, PA can be divided into about twenty serotypes based on the variations of O antigen, and there are a large number of variations among PA genomes^34^. One strategy for PA vaccine development is to identify and test conserved proteins as vaccine antigens to achieve broad protection. Among all the possible PA antigens, PcrV, OprF, and OprI are key, since they are expressed in the majority of the pathogenic PA strains and play vital roles in the pathogenesis of PA^46,47^. PcrV, the extracellular component of T3SS, which plays a major role in the development of pathogenicity via killing the epithelial and immune cells by injecting a toxins^12,13^. OprF anchors the outer membrane of PA peptidoglycan layer and is the crucial protein to induce the expression of full virulence^48^. OprI is a key lipoprotein for the resistance of the PA against antimicrobials and is characterized by antigenic cross-reaction with all the serotype strains^49^. PcrV is highly conserved and is expressed in about 90% of clinically isolated strains. The outer membrane proteins OprF and OprI are highly conserved and serotype-independent that have indicated great performance in several studies although a phase III clinical trial employing a hybrid fusion protein of OprF and OprI (IC43) has failed^11^. Several studies have shown that active immunization with PcrV, OprF, and OprI could provide protection to some degree against PA infection. However, Previous studies have revealed that protein subunit vaccines do not elicit enough protection from infections^50–52^. In this study, two mRNA vaccines encoding PcrV and OprF-I fusion protein, respectively, were constructed, expecting to achieve the cross and adequate protective effect. For the optimization of immunization outcome, the mRNA vaccines were nucleoside modified, particularly, uridine triphosphate was replaced by N1-methyl-pseudouridine (m1Ψ) triphosphate. Nucleoside modification aids in the augmentation of translation efficacy, stabilizes the mRNA^53^, and induces a strong and long-term cellular immune response^33^. LNP is the optimal delivery system for mRNA vaccines. Indeed, they are successfully used as excellent carrier platforms for current SARS-CoV-2 mRNA vaccines^41,42^. A number of studies have tried to explore the mechanism for the clearance of *P. aeruginosa*^34^, but there is still no clear understanding of what kind of immune response is most important for an effective PA vaccine. Some studies highlighted the importance of humoral immune responses, and proposed that a Th1-predominant immunity is optimal for the prevention of PA infection^54–57^. In recent years, several studies have demonstrated that the Th17 immune response is required for protection against PA infection^8,34,58^. In the current study, both PcrV and OprF-I mRNA vaccines elicited combined humoral and cellular immune responses, accompanied by mixed Th1/Th2 responses. The protective Th1-biased immune response induced by the mRNA-PcrV-LNP and mRNA-OprF-I-LNP in this study is consistent with other studies showing that Th1-biased immunity is protective against P. aeruginosa respiratory infections^59,60^.

Notably, although both mRNA-PcrV-LNP and mRNA-OprF-I-LNP vaccines could induce immunity, the PcrV vaccination elicited a higher level of humoral and cellular immune responses and significantly reduced the colonization of bacteria in the organs, providing a broad protective effect against PA infection. The immune response and protection elicited by mRNA vaccines separately and mixed together showed the superiority of mRNA vaccines over PcrV and OprF-I protein vaccines. Despite the stronger humoral immunity induced by mRNA-PcrV-LNP, immunization with mRNA-OprF-I-LNP demonstrated only slightly lower OPK capacity compared with mRNA-PcrV-LNP group. This can be explained by the difference between the levels of produced Ig types in these two experimental groups. Glycosylation also affects the function of the Igs^61^. The OPK capacity may also be dependent on antibody avidity while the avidity is not always positively correlated to the IgG concentration^62^. In conclusion, our research is the first attempt to apply mRNA vaccine technology for developing PA vaccine. The results suggested that PcrV encoding mRNA vaccine, as well as combined mRNA-PcrV + mRNA-OprF-I vaccine seems to be promising vaccine candidates to prevent PA infection.

## Methods

### Mice and strains

Specific pathogen-free female 4–6 weeks old BALB/c mice (20-30 g) were purchased from Kangde Biological (Guangzhou, China). All animal experiments and research complied with relevant ethical regulations, and the research has been ethically approved by the Animal Experiment Center of Southern University of Science and Technology and the Shenzhen Research Institute of Hong Kong Polytechnic University. *P. aeruginosa* standard strain PAO1 (ATCC^®^47085^TM^) was purchased from China Culture Collection Center. PA strains PA16, PA117, and PA257 were obtained from the research group of Professor Yang Liang, Department of Microbiology and Immunology, Southern University of Science and Technology (Shenzhen, China).

### Design and synthesis of PcrV mRNA vaccine and OprF-I mRNA vaccine constructs

PcrV, OprF, and OprI protein sequences were retrieved from NCBI (Uniport: PA1706). As a secretion signal peptide, the leader sequence of tissue plasminogen activator (TPA) was added at the N-terminus, while the 6*His tag was adjoined at the C-terminus of the construct. For the further optimization of the vaccine sequence, 5’ and 3’ untranslated regions (UTRs) of human beta globulin were added. Additionally, the Kozak sequence at the 5’ end of the ORF and a polyadenylation (PolyA) sequence at the 3’ end were adjoined. The above gene fragments were synthesized and cloned into the pVAX1 vector to obtain template plasmids pVAX1-PcrV and pVAX1-OprF-I.

### Plasmid template amplification and linearization

The pVAX1-PcrV plasmid and pVAX1-OprF-I plasmid were transformed into DH5α competent cells via the heat-shock method. After amplification, bacterial cells were cultured on kanamycin-resistant Luria-Bertani (LB) plates and incubated at 37 °C for 12 to 15 h. The single colony was then picked and added to the LB broth containing Kanamycin in a conical flask, placed in an incubator at 37 °C for 12 to 15 h, while shaken at 250 r/min. Bacterial cells were collected by centrifugation, according to the instructions of the plasmid DNA extraction kit, and plasmid extraction was performed on the obtained bacterial cells (Vazyme, Nanjing, China). For the linearization, the enzyme XhoI cleavage site of the amplified plasmid was selected next to the PolyA tail. The degree of linearization was detected by DNA agarose gel electrophoresis. The linearized plasmid DNA was recovered by PCR product recovery kit (Thermo Fisher Scientific, US), and the DNA concentration and quality were determined by NanoDrop 2000c (Thermo Fisher Scientific, US). The OD260 and OD260/OD280 values were determined by the nucleic acid analyzer (Thermo Fisher Scientific, US).

### In vitro transcription and purification of mRNA

In vitro mRNA synthesis was performed via T7 polymerase-mediated DNA transcription. The steps of in vitro transcription IVT are as follows: (1) RNase-free water and NTPs were mixed; (2) CleanCap® Reagent AG (3’ OMe) was added, mixed, and centrifuged; (3) 10×Transcription Buffer was added to the solution, mixed, and centrifuged, the supernatant was collected; (4) DNA template was added; (5) Murine RNase Inhibitor, Yeast Inorganic Pyrophosphatase, and T7 RNA Polymerase were mixed well with the solution and centrifuged, supernatant to was collected; (6) After the reaction at 37 °C for 2-3 h, DNAaseI was added to remove the template DNA, and the transcript was purified by Monarch RNA cleanup kit (Thermo Fisher Scientific, US). The concentration of mRNA was calculated from readings by NanoDrop 2000c UV spectrophotometer, and the purity of mRNA was determined by capillary electrophoresis 2100 Bioanalyzer.

### mRNA-LNPs preparation

The specific preparation process of LNP is as follows: dissolving ionizable lipid (DLin-MC3-DMA), cholesterol, auxiliary lipid (DSPC), and polyethylene glycol in absolute ethanol according to the ratio of 50:38.5:10:1.5; The total concentration of 10 mg/ml was used to form the organic phase; The mRNA encoding the antigenic protein was diluted in sodium citrate buffer (50 mM, pH 4) to a final concentration of 0.1 mg/ml to form an aqueous phase; the organic phase was mixed with water with a ratio of 1:3 using the microfluidic device (Inano E, Micro&Nano, Shanghai, China) at a speed of 12 ml/min to obtain the mRNA-LNP mixture; The mRNA-LNP mixture was then diluted 40-fold with sterile PBS (10 mM, pH 7.2) and transferred to a pre-sterilized Amicon® Ultra-15 centrifugal filter (cutoff = 10 KDa). For buffer exchange and target sample concentration, centrifugation was done at 4000 × *g* for 15-30 minutes at 4 °C before adding fresh PBS. This was repeated 3 times to obtain mRNA-LNP at a concentration of 2 mg/ml. The last sample was stored at 4 °C and used within a week.

### Determination of particle size, uniformity, and encapsulation efficiency of mRNA-LNPs

The particle size and uniformity of LNPs were measured by dynamic light scattering (DLS) instrument (Mastersizer 3000, Malvern Panalytical, United Kingdom). The concentrated sample was diluted at 1:100 in sterile PBS, and HORIBA-SZ100 equipment was used at dispersion angles of 25° and 90°. This was repeated three times to obtain the particle size distribution and polymer dispersity index (PDI) value of LNP. Particle size results were given as the ratio of particle size to strength and also to predict the stability of the LNP dispersion system. The encapsulation efficiency of mRNA was determined by Quant-iT ™ RiboGreen ™ RNA kit (Invitrogen, R11490). The measurement principle is as follows: Quant-iT ™ RiboGreen ® RNA reagent is an ultra-sensitive fluorescent nucleic acid stain, which can detect 1-200 ng of nucleic acid in solution. This nucleic acid dye cannot penetrate LNP and only free nucleic acid that is not encapsulated by LNP can be detected. Triton-100 is often used as a surfactant and a demulsifier. LNP-mRNA obtained by treating with 1% Triton-100 can release the encapsulated nucleic acid and obtain the total nucleic acid amount. The drug loading amount is obtained by calculating the difference in the amount of nucleic acid before and after demulsification, and then dividing by the total nucleic acid amount to obtain the encapsulation efficiency: Encapsulation efficiency (%) = (quantity after demulsification - quantity before demulsification) / quantity after demulsification.

### mRNA-LNPs cytotoxicity assay

HEK 293 T cells were adherently cultured and different doses of mRNA were added to test the cytotoxicity of mRNA-LNPs. Briefly, 4-6 h after seeding cells in 96-well plates, the dose of 25 µg mRNA LNPs was added. Twenty-four to seventy-two hours after the addition of high and low doses of mRNA-LNP, 10 µl of CCK8 (Vazyme, Nanjing, China) was added to each well and cultured for 0.5-4 h. The number of cells per well was quantified by measuring the OD at 450 nm with a microplate reader.

### mRNA and mRNA-LNPs transfection, Western Blot

Cell transfection of mRNA-PcrV and mRNA-OprF-I was performed via both LNP and lipofectamine 3000 (Thermo Fisher Scientific, US). The transfection of HEK293T cells with mRNA-LNP was done by adding mRNA-PcrV-LNP and mRNA-OprF-I-LNP to the cells of ~70% confluency with the mRNA concentration of 0.5 µg/cm^2^. The transfection with lipofectamine 3000 was carried out according to the manufacturer’s instructions. Stably growing HEK293T cells were plated in six-well plates and were transfected at 70-80% confluency. 4 μg of mRNA-LNPs were diluted in 200 μl Opti-MEM (Gibco, US), and incubated with 10 μl of lipofectamine 3000 (also diluted in 200 μl Opti-MEM) for 15 min at room temperature. The mixtures were then added to the cell culture media and incubated for 48 h. The cell culture supernatants and cell lysates were then obtained and the expression of mRNA-PcrV, mRNA-PcrV-LNP, mRNA-OprF-I, and mRNA-OprF-I-LNP was analyzed by Western blot (WB). Samples were mixed with 5x loading buffer, boiled for 10 min, separated by SDS-polyacrylamide gel electrophoresis, and electrotransferred onto polyvinylidene fluoride (PVDF) membranes using the eBlot^TM^ L1 fast wet rotator (GenScript, Nanjing, China). Membranes were blocked with 1% bovine serum albumin (BSA) for 1 hour and washed 3 times with 0.1% Tween Phosphate Buffered Saline 0.1% Tween (PBST) buffer. The PcrV and OprF-I proteins were detected by incubating the membranes with an anti-His-HRP antibody (dilution 1:10.000) (HRP-66005, Proteintech, US) for 1.5 h at room temperature and then visualized via Tanon 5200 chemiluminescent imaging system.

### Expression and purification of target proteins

PcrV and OprF-I protein-encoding genes were cloned into a pET28a expression vector (Invitrogen, US) with a C-terminal 6×His tag and transformed into BL21(DE3) pLysS competent cells. A single colony carrying the target protein particles expressed recombinant PcrV and OprF-I proteins carrying 6 consecutive histidine residues under IPTG induction at 37 °C. The cells collected by centrifugation were crushed using a high-pressure homogenizer, and the supernatant obtained after high-speed centrifugation was firstly purified by NTA-Ni affinity chromatography (GE, US), and then by size-exclusion chromatography (SEC) (Cytiva, US).

### Immunization of burn and systemic infection mouse models

For in vivo assessment of immune responses induced by mRNA and protein vaccines, BALB/c mice were immunized via intramuscular injection of 100 µL mRNA-PcrV-LNP (*n* = 8), mRNA-OprF-I-LNP (*n* = 8), mRNA-PcrV + mRNA-OprF-I –LNP (*n* = 8), blank LNP (*n* = 8), PcrV protein (*n* = 8), OprF-I protein, and PcrV + OprF-I protein (*n* = 8) mixture. Aluminum hydroxide adjuvant (Thermo Fisher Scientific, US) was added in the protein vaccines with the ratio of 1:1 (vol:vol). There were two different immunization doses were administered: a low–dose of 5 µg and a high dose of 25 µg. The second booster immunization with the same dose and volume was performed 3 weeks after the first immunization. Blood samples were collected from the orbital venous plexus on the 1st, 3rd, and 5th weeks after the primary vaccination, and the serum was obtained for further analyses.

The burn mouse model was developed as follows: All animals (immunized and unimmunized groups) were first anesthetized with 250 μL of 2.5% Avertin, then the right side of the waist along with the back of the body with a final diameter of 22 mm and the length of 100 mm was depilated with a depilatory cream. A metal block weighing 165 g was heated to 104 °C and was applied to the depilated site of the mice for 8 s to induce a 3rd-degree burn. Immediately thereafter, mice received intraperitoneal injections of 500 μL of 0.9% saline and 40 μL of meloxicam (1 mg/kg) every 24 h to prevent irritation and pain. Ultimately, 10×LD50 of different PA strains were injected under the burned skin. For mouse systemic infection models, we infected mice systemically with 10×LD50 bacteria via tail vein injection. The timeline of immunization, blood collection, and burn induction is shown in Fig. 3.

### Determination of mouse-specific antibody titers and antibody typing via ELISA

The specific antibody titers in the sera were determined by indirect enzyme-linked immunosorbent assay (ELISA). The steps are as follows: The blank ELISA plate was coated with PcrV and OprF-I expressed in *E. coli* and incubated at 4 °C overnight; After washing 3 times with PBST, 1% BSA was added to each well and blocked at 37 °C for 2 h; After washing 3 times, 100 μl of serially diluted mouse sera were added to each well, incubated 37 °C for 60 min, and gently shaken; After 3-times washing, 100 μl of HRP-labeled rabbit anti-mouse IgG (diluted 1:1000) was added and incubated at 37 °C for 60 min for the reaction, gently shaken; The wells were washed 3 times, 100 μl of HRP chromogenic substrate TMB was added and reacted at room temperature for 15 min in the dark; ultimately, 100 μl of 2% sulfuric acid was added to stop the reaction; OD450 detected absorbance. As the primary antibodies, anti-IgG1 (dilution 1:20,000) (SA00012-1, Proteintech, Manchester, UK), IgM (dilution 1:20,000) (SA00012-6, Proteintech, Manchester, UK), and IgG2a (dilution 1:20,000) (SA00012-2, Proteintech, Manchester, UK) were used while an HRP-labeled goat anti-mouse antibody (dilution 1:20,000) (ab6789, Abcam, Cambridge, UK) was used as the secondary antibody.

### Intracellular flow cytometry analysis and ELISpot Assay

Mice were sacrificed 14 days after the 2nd immunization, the spleens were taken immediately after the euthanasia. After grinding the spleen, it was washed with PBS and passed through a 200-mesh cell strainer to obtain a single-cell suspension. After adding 3 mL of red blood cell lysate to lyse, the lymphocytes were obtained by washing twice with sterile PBS, counted with a cell counter, and inoculated 2 × 10*6 cells in each well of a cell culture 6-well plate. Then different peptides (2 μg/ml) were added to stimulate for 6 hours, and the inhibitor Brefeldin A was added for another 6 h of incubation. Washing was performed with PBS with 0.2% BSA, resuspended in 100 µl, and the cells were incubated with the specific antibody CD16/CD32 for half an hour. The centrifugation and washing were repeated 3 times, and the final resuspension was done quantitatively. All experimental groups were added with fluorescent dye-conjugated monoclonal Antibodies- anti-mouse APC-CD3 (dilution 1:50) (100248, Biolegend, San Diego, California, US), antimouse FITC-CD4 (dilution 1:50) (100510, Biolegend, San Diego, California, US), and anti-mouse BV510-CD8 (dilution 1:100) (100752, Biolegend, San Diego, California) were incubated for 30 minutes.

In the next step, intracellular cytokine staining was performed, and the cells were permeabilized and fixed with Cytofix/Cytoperm Fixation/Permeabilization Kit (BD Biosciences, US). Mouse IFN-γ and PE/Cy7 anti-mouse IL-2 were incubated in the dark at 4 °C for 30 min. After the final centrifugation samples were washed and transferred to the flow loading tube by flow tube filtration before loading on the machine. All the above antibodies were purchased from Biolegend (San Diego, CA). Samples were analyzed on a BD FACS Array flow cytometer (BD Biosciences, US) using BD FACSArray software™.

The lymphocyte suspension was obtained by processing according to the method used above. After counting by a cell counter, 5 × 10*5 cells were plated in each well, and the same polypeptide and concentration mentioned above were added to stimulate for 24 h. It was followed by washing with PBS 5 times, adding 100 µl of diluted 1 µg/mL antibody, and incubation at room temperature for 2 h. After repeated washing, streptavidin-HRP secondary antibody (dilution 1:000) (3321-4HST-2, Mabtech, US) was added for 1 h at room temperature. It was washed and the residual liquid was absorbed, TMB color-developing solution was added, and the assay was protected from light for 5-10 minutes. The liquid was blotted dry, washed with deionized water to stop the reaction, and finally placed in the dark, waiting for the plate to be read. The above experiments were carried out according to the instructions of the mouse IFN-γ ELISpot PLUS kit (Mabtech, US).

### Opsonophagocytic killing assay

HL-60 cells (ATCC, CCL-240) were first cultured for differentiation, and after 5 days in 100 mM N’N-dimethylformamide in IMDM medium, they were differentiated into granulocyte-like cells and treated with calcium and magnesium-free and calcium-magnesium-containing HBSS buffer and finally resuspended in conditioning buffer. At the same time, we heat-inactivated the mRNA vaccine immune serum samples. Add 40 µl of 4×10*5 HL-60 cells to each well of a 96-well plate, 10 µl of 10*3 CFUs of standard strain PAO1 or clinical strains PA16, PA117, PA257 in opsonophagocytic buffer, and finally, 20 µl immune serum and 10 µl 1% guinea pig serum were added in each well as a complement source. After 2 h in the cell incubator, 10 µl of each sample was inoculated onto agar plates for overnight colony culture. The opsonophagocytic killing effect of the vaccine-immunized sera was demonstrated by the reduction ratio of colony-forming units (CFU) in the experimental group compared with the colony-forming units in the serum of unimmunized mice.

### Evaluation of bacterial load

After 24 h of infection of mice at a dose of 10 × LD50 of each type of bacteria, the mice were observed, sacrificed, and the organs were separated and weighed. The isolated skin, lung, liver, spleen, and kidney samples were triturated, homogenized, and labeled in 4 mL of sterile PBS. Then, the samples were serially diluted with sterile PBS, and 50 µl of the diluted samples were placed on a solid medium plate and incubated overnight in a bacterial incubator. Colony counts were performed the next day and the number of CFUs per sample was calculated in grams of tissue (CFU/g).

### Antibiotic resistance analysis

Bacterial resistance of *P. aeruginosa* strains including PAO1, PA16, PA117, and PA257 was measured according to the guidelines of the Clinical and Laboratory Standards Institute (CLSI, 2016). Based on the size of the inhibition circle, bacteria are classified as resistant (R), intermediate (I), or susceptible (S) to antibiotics^63^.

### ExoU determination

ExoU of *P.aeruginosa* strains PAO1, PA16, PA117, and PA257 was measured by sandwich ELISA. The kit (MK617A) was purchased from Lianhui Experimental Instrument Co., Ltd., China. The microtiter plate wells were pre-coated by purified capture antibody. After the immobilization, same concentrations of bacterial lysate solution were added to wells that was followed by adding the anti-ExoU detection antibody (HRP-labeled). The antibody–antigen–antibody (HRP) complex was generated. After washing the plate, TMB substrate solution for visualizing HRP enzymatic reaction was added. TMB was catalyzed by HRP and the reaction was terminated by the addition of a sulfuric acid solution and the color change was measured spectrophotometrically at a wavelength of 450 nm. The concentration of ExoU in the samples was then determined by comparing the O.D. of the samples to the standard curve.

### Ethics statement

All animal studies are approved by the Animal Ethical and Experimental Committee of the Southern University of Science and Technology and The Hong Kong Polytechnic University (NO.22-23/286-OTHERS-R-SZG).

### Statistical analysis

The data are presented as the mean ± SE. The significance of differences was determined by an unpaired parametric test (Student’s t-test for two groups or one-way ANOVA for more than three groups). For pairwise comparisons among three or more groups, *p* values were adjusted by using Tukey’s test. SPSS15.0 and GraphPad Prism 8.0 were used for data analysis. The data were considered significant when the *p* value was < 0.05; *P < 0.05; **P < 0.01; ***P < 0.001; ns, not significant. Two-tailed Student’s t-test was used to determine the P value between two groups. The data for survival test were analyzed by Wilcoxon log-rank survival test (**P < 0.01).

### Reporting summary

Further information on research design is available in the Nature Research Reporting Summary linked to this article.

## Supplementary information

Supplementary Materials

REPORTING SUMMARY

## Data Availability

All data are available in the main text or Supplementary information. Additional information is available from corresponding authors upon request.
