# Supplementary material for: Strong immune responses and protection of PcrV and OprF-I mRNA vaccine candidates against Pseudomonas aeruginosa
Source: NPJ Vaccines. 2023 May 25;8:76. doi: 10.1038/s41541-023-00672-4 (PMC10209580; doi:10.1038/s41541-023-00672-4)
Supplement: Supplementary file 1 — Supplementary Materials [file 41541_2023_672_MOESM1_ESM.pdf]

Supplementary information

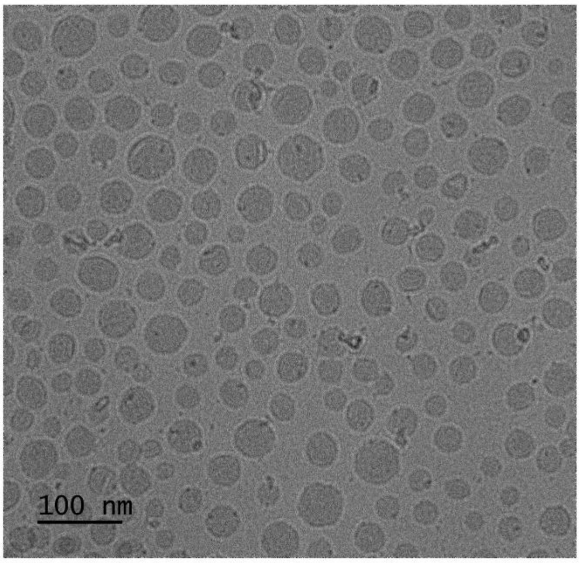

Supplementary Figure 1. Cryo-TEM images of LNP wrapped mRNA (right) (Scale-bar, 100 nm).

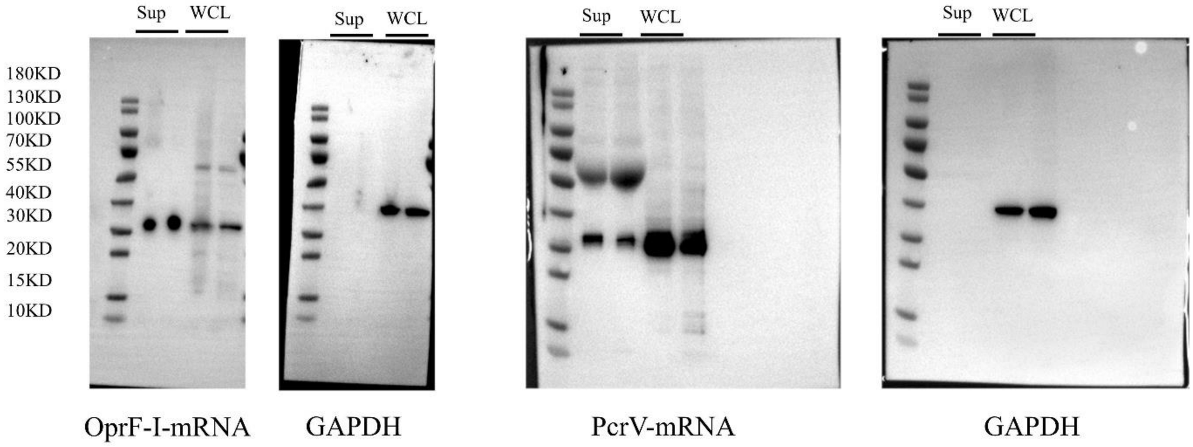

Supplementary Figure 2. Original blots of proteins– OprF-I-mRNA, PcrV-mRNA, and GAPDH.

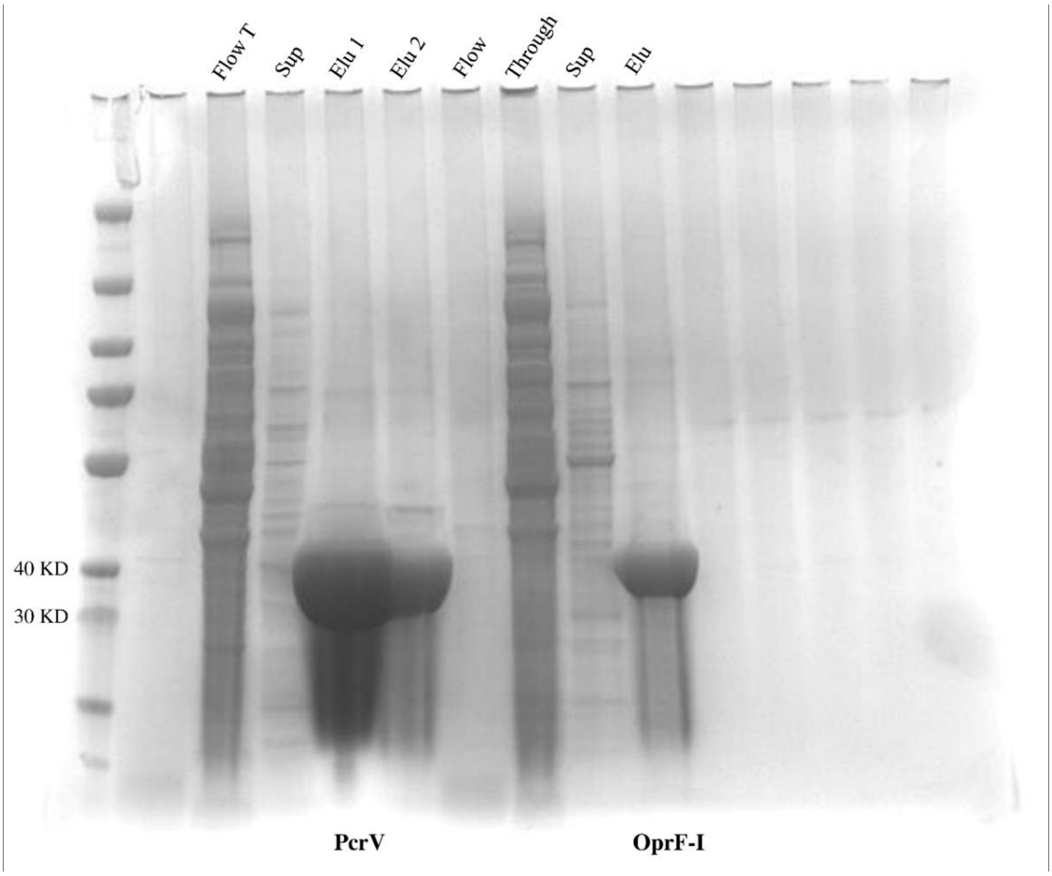

Supplementary Figure 3. SDS-PAGE gel of purified PcrV and OprF-I proteins by Ni-NTA affinity column.

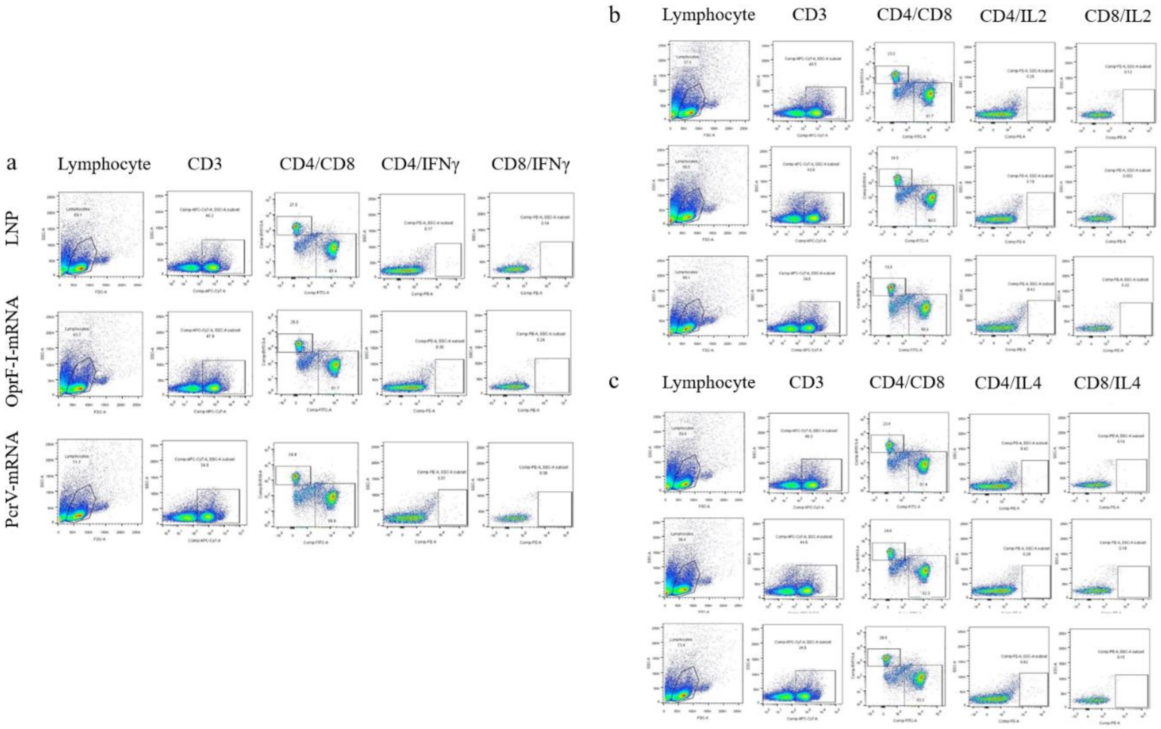

Supplementary Figure 4. Gating strategy for surface and intracellular staining flow cytometry. (a) Gating strategy to sort CD4+ cells expressing IFN-γ and CD8+ cells expressing IFN-γ in different groups. (b) Gating strategy to sort CD4+ cells expressing IL-2 and CD8+ cells

expressing IL-2 in different groups. (c) Gating strategy to sort CD4+ cells expressing IL-4 and CD8+ cells expressing IL-4 in different groups.

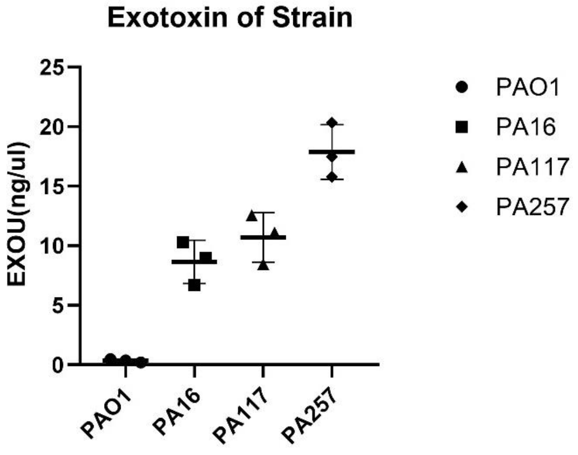

Supplementary Figure 5. Exotoxin analysis of the PA strains used in this study.

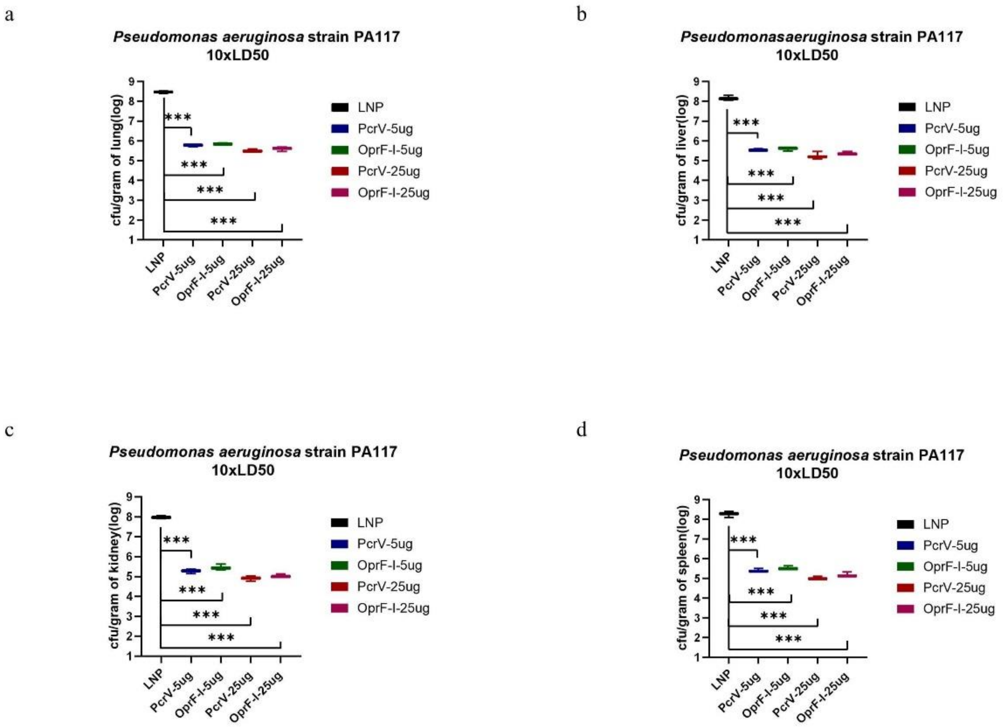

Supplementary Figure 6. PA PA117 strain loads in the lung (a), liver (b), spleen (c) and kidney (d) of vaccines or LNP immunized mice 24 h after challenging with the 10xLD50 CFU in systemic infection model. *p< 0.05, **p< 0.01 and ***p< 0.001. Abbreviation: ns, not significant.

Supplementary Table 1: Peptide sequences used in Elispot and flow cytometry assay.

**PcrV-Peptide**	AHAGQPLSEAQVLKALAW
	DLVDPTLYGYAVGDPRWK
	LLALLRSERIVLAHAGQP
	RQPGAQWDLREFLVSAYF
	SRPLNDKVNEKTTLLNDT
**OprF-I-Peptide**	DVKFDFDKSKVKENSYADIK
	DSVGTDAYNQKLSERRANAV
	RLTATEDAAARAQARADEA
	DEALGAAQKAQQTADEAN

Supplementary Table 2. The median-lethal dose of PAO1 and PA257 strains in burn model.

**Strains**	**$LD_{50(CFUs)}$**	**$10XLD_{50(CFUs)}$**
PAO1	1X10*4	1X10*5
PA257	0.9X10*4	9X10*4

Supplementary Table 3. The median lethal dose of PAO1, PA16, and PA117 strains in a systemic infection model.

**Strains**	**$LD_{50(CFUs)}$**	**$10XLD_{50(CFUs)}$**
PAO1	2X10*6	2X10*7
PA16	1X10*6	1X10*7
PA117	8X10*6	8X10*7

Supplementary Table 4: Antibiotic resistance of PA strains used in this study.

**Strains/Antibiotic**	**Penicillin**	**Streptomycin**	**Gentamicin**	**Norfloxacin**	**Ciprofloxacin**	**Cefoperazone**
PAO1	0	22mm	24mm	24.6mm	30mm	32mm
	R	S	S	S	S	S
PA16	0	21.8mm	21.6mm	42mm	44mm	24mm
	R	S	S	S	S	S
PA117	0	0	0	18.6mm	19.8mm	0
	R	R	R	S	I	R
PA257	0	18mm	18.6mm	10.2mm	12mm	18.8mm
	R	S	S	R	R	I

Notes: Different categories of antibiotic susceptibility testing are abbreviated as S– susceptible, I– intermediate, and R– resistant.

Supplementary Table 5: Clinical background of PA isolates.

Strains	Age	Source	Date
PA16	15	Peritoneal drainage fluid	2013.12.12
PA117	56	Urine	2014.01.19
PA257	48	Sputum	2014.03.07

Supplementary Note 1

mRNA-PcrV ORF encoded protein sequences:

MDAMKRLCCVLLLCGAVFVSPMEVRNLNAARELFLDELLAASAAPASAEQEEL
LALLRSERIVLAHAGQPLSEAQVLKALAWLLAANPSAPPGQGLEVLREVLQARRQPG
AQWDLREFLVSAYFSLHGRLDEDVIGVYKDVLQTQDGKRKALLDELKALTAELKVY
SVIQSQINAALSAKQGIRIDAGGIDLVDPTLYGYAVGDPRWKDSPEYALLSNLDTFSG
KLSIKDFLSGSPKQSGELKGLSDEYPFEKDNNPVGNFATTVSDRSRPLNDKVNEKTTL
LNDTSSRYNSAVEALNRFIQKYDSVLRDILSAIGGGSHHHHHH

mRNA-OprF-I ORF encoded protein sequences:

MKLKNTLGVVIGSLVAASAMNAFAAPAPEPVADVCSDSDNDGVCDNVDKCPD
TPANVTVDANGCPAVAEVVRVQLDVKFDFDKSKVKENSYADIKNLADFMKQYPSTS
TTVEGHTDSVGTDAYNQKLSERRANAVRDVLVNEYGVEGGRVNAVGYGESRPVAD
NATAEGRAINRRVEGSGSGSGSGSHSKETEARLTATEDAAARAQARADEAYRKADEA
LGAAQKAQQTADEANERALRMLEKASRKGGGSHHHHHH

Red color denotes signal peptide, green color– PcrV protein sequence, light blue– linker, purple– polyhistidine tag, blue– OprF protein sequence, orange– OprI protein sequence.
